# Protein-Dependent, pH-Selective Complexation in Tragacanth–Protein Systems: An Integrated FTIR–DLS–Rheology–Docking Study

**DOI:** 10.3390/ijms262311333

**Published:** 2025-11-24

**Authors:** Jagoda O. Szafrańska

**Affiliations:** Department of Dairy Technology and Functional Food, Faculty of Food Sciences and Biotechnology, University of Life Sciences, Skromna 8, 20-704 Lublin, Poland; jagoda.szafranska@up.edu.pl

**Keywords:** protein–polysaccharide complexes, tragacanth gum, pH-dependent complexation, FTIR, DLS, rheology, molecular docking

## Abstract

Tragacanth gum (GT) was mixed with whey protein concentrate (WPC80), whey protein isolate (WPI) or rice protein (RP) across pH 3.0–7.2 in order to clarify the effect of protein type and pH on controlling association and bulk behavior. Turbidimetry at 600 nm by photographic validation, oscillatory and steady-shear rheology, dynamic light scattering (DLS), FTIR spectroscopy, and AutoDock Vina docking were employed and compared. Whey systems reflected a clear, mildly acidic window: low-strain elasticity (G′) reached near pH ~5, with increased A600 and dominant sub-100 nm DLS modes, reflecting associative complexation near the isoelectric region. WPI also reflected a secondary turbidity/viscosity rise at pH 7.2, consistent with segregative aggregationafter the associative window. RP was variable, featuring broadly increased turbidity with viscosity/DLS maxima at pH 6.4, reflecting glutelin-facilitated solubility/aggregation rather than an acid optimum. FTIR changes in the amide band and GT bands (COO^−^ ~1400–1406 cm^−1^; 1015–1040 cm^−1^) supplemented enhanced coupling at pH 3–5. Superimposition through docking of multivalent hot-spots (Lys/Arg and H-bonding neighborhoods) corresponded to the phase-level readouts. Together, the data establish protein-dependent, pH-selective windows for GT–protein systems and uncover a mechanistic dichotomy: associative complexation in whey vs. neutral-side, solubility-regulated aggregation in RP.

## 1. Introduction

Recent research has indicated that the interaction between tragacanth gum and various protein types can significantly influence the functional properties of the resulting biopolymer systems [1,2]. Furthermore, the pH of the medium in which these interactions occur plays a crucial role in modulating the rheological behavior of these systems [1,3]. The functional properties of tragacanth gum, such as viscosity, gel formation, and emulsification, were found to be significantly affected by the type of protein incorporated into the system [1,2,4,5]. In addition to protein type, pH is a critical factor governing the functional properties of tragacanth gum–protein systems [1,4,6,7]. Because protein net charge and solubility are pH-dependent, changes in pH alter electrostatic attraction with the anionic tragacanth gum and thereby modulate viscosity, gelation, and coacervation behavior [8,9]. At lower pH (near or below a protein’s isoelectric point), proteins tend to be positively charged, which strengthens complexation with tragacanth gum and can increase viscoelasticity/gel strength [1,4,7]. At higher pH, proteins become neutral or negatively charged, reducing affinity and weakening the network [6,7]. Altogether, these findings underscore the need to optimize both protein type and pH to achieve the desired functionality in tragacanth gum–protein systems [1,4,7].

The rheological properties of tragacanth gum–protein systems are strongly governed by the interplay between protein type and pH. For whey protein isolate (WPI), sodium caseinate, pea protein isolate (PPI) and sesame protein isolate (SPI), changes in pH modulate electrostatic attraction to the anionic tragacanth gum, which in turn drives viscosity, yield stress and gel/solid-like responses [1,4,6,7]. Specifically, near or below a protein’s isoelectric point (lower pH), proteins carry a positive net charge, leading to stronger complexation/coacervation with TG and higher viscoelastic moduli; at higher pH, the affinity weakens and the structure softens [4,6,7]. Practical food systems show the same trend: adding TG to WPI-stabilized emulsions or to dairy matrices modifies flow behavior and yield stress through protein–TG networking [10,11,12]. These results underscore that choosing the protein type and tuning pH are both essential to target viscosity, yield stress and flow behavior in TG–protein formulations [1,4,7]. The implications of these findings extend well beyond academia, with clear industrial and biomedical relevance. By selecting protein type and tuning pH, formulators can steer the viscosity, yield stress, and gel/solid-like behavior of tragacanth gum–protein systems to create stable food emulsions, edible/active films, and biomedical dressings [1,4,6,7]. In foods, TG combined with whey protein isolate or caseinate improves emulsion stability and modulates flow properties/yield stress. TG–protein networking under appropriate pH windows is key [10,11,13]. TG also forms edible/active films with proteins (e.g., WPI, gelatin, soy protein) that show enhanced mechanical/barrier performance attributes, which are valuable for packaging and delivery of actives [2,5,14]. In pharmaceutical/biomedical contexts, TG–gelatin composites and TG-based hydrogels are explored for drug delivery and wound healing, where protein–TG interactions, strengthened at suitable pH, support film integrity, bioactivity, and stability [8,15,16]. Altogether, protein choice + pH control provide a practical toolbox to tailor TG–protein systems for novel food products and pharma-grade biomaterials [8,17].

In conclusion, studies consistently show that the functional and rheological properties of tragacanth gum–protein systems arise from a coupled effect of protein type and pH [1,4,6,7]. Selecting proteins that complex/coacervate strongly with the anionic TG at appropriate pH near the protein’s pI or within the coacervation window enables targeted tuning of viscosity, yield stress, and gel-like behavior [1,4,7]. These insights translate directly to practice: TG–protein interactions have been leveraged to stabilize food emulsions and to adjust flow properties in dairy and emulsion systems [10,11], and to engineer films/biomaterials for packaging and wound-care/drug-delivery applications [2,5,8,17]. As research progresses, further mechanistic details will likely expand these industrial and biomedical applications of TG–protein biopolymer systems.

This work tests the hypothesis that pH-selective associative complexation between an anionic, uronate-rich polysaccharide (tragacanth, GT) and proteins creates protein-dependent formulation windows in structure and functionality. Specifically, whey proteins (WPC80, WPI) are expected to show a mildly acidic optimum near the protein isoelectric region, whereas rice protein (RP), dominated by glutelin, should exhibit neutral-side behavior governed by solubility/aggregation, rather than a sharp acidic maximum. To link molecular signatures with bulk outcomes, FTIR (ion-pairing/H-bonds), DLS (dispersion state), oscillatory and steady-shear rheology (elasticity and flow), and AutoDock Vina (multivalent hot-spots on the protein surface) are interpreted jointly, providing an integrated basis for mapping these windows. Consequently, whey proteins (WPC80, WPI) are contrasted with RP to expose protein-type effects. The study is application-driven, aiming to identify pH ranges that favor either cohesive, gel-like structure (for texture/structuring) or high viscosity with limited elasticity (for thickening) in foods and protein–polysaccharide films/biomaterials. Its novelty lies in a single, integrated protocol that jointly maps FTIR markers, nanocomplex size, and bulk mechanics across three protein classes, it reveals a mechanistic dichotomy-associative complexation in whey versus neutral-side, solubility-regulated aggregation in RP and provides practical pH windows (acidic for GT–whey; neutral-side for GT–RP) as immediately usable formulation guidance.

WPC80 and WPI were selected as globular whey proteins that carry positively charged patches near their isoelectric region, making them model systems for associative complexation with an anionic polysaccharide. Rice protein (RP), dominated by glutelin storage globulins with low aqueous solubility and higher aggregation propensity in the neutral range, was included to represent a solubility/aggregation-controlled contrast to whey. This set enables direct comparison of shared spectroscopic features (amide bands, GT fingerprint) with divergent dispersion/mechanics driven by pH-dependent charge and solubility.

## 2. Results

### 2.1. Turbidity Measurements

**Figure 1:**
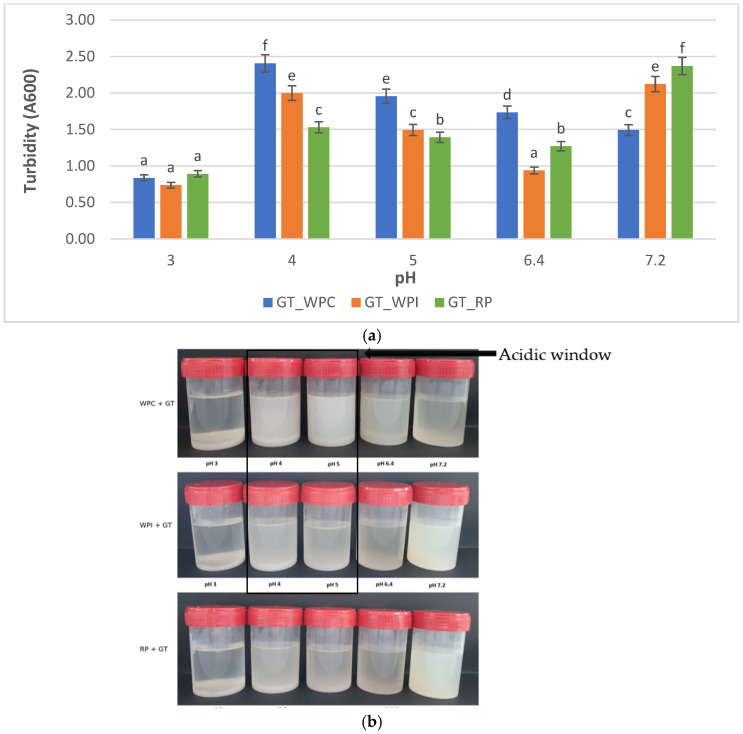
(**a**) Turbidity (A600) of GT–protein mixtures (WPC80, WPI, RP) versus pH. Different letters indicate significant differences among pH points within each protein system. (**b**) Visual appearance of GT–protein mixtures at different pH values (3.0–7.2).

For all GT–protein systems, turbidity at 600 nm (A600) and visual appearance follow the same pH-dependent patterns (Figure 1a,b, Table 1).

Turbidity peaks at pH 4–5 for the GT–WPC samples (A600 = 2.404 and 1.956) and decreases toward neutral pH (7.2: 1.492). The photographs show the same trend: the pH 4–5 vials are the most opaque, whereas pH 3 and 7.2 appear noticeably clearer. This is the classic acidic window of associative protein–polysaccharide complexation near the whey–protein isoelectric region [4,18]. For the GT–WPI samples, A600 is high at pH 4 (1.998) and remains elevated at pH 7.2 (2.121), with minima at pH 3–6.4 (0.728–0.938). The photo panel mirrors these values. The acidic maximum around pH 4 aligns with coacervation near pI, while the neutral-pH rise suggests aggregate growth/segregated structuring specific to this composition/ionic strength, in line with prior reports for whey–polysaccharide systems [4,19]. In contrast, for the GT–RP samples, turbidity is highest at pH 7.2 (2.368) and lower across pH 3–6.4 (0.891–1.529); visually, all vials look milky, with pH 7.2 the most opaque. This broad, high-turbidity baseline matches rice glutelin’s poor solubility and strong aggregation propensity outside extreme pH, sustaining large scattering particles even where purely electrostatic attraction to GT is reduced [20]. Thus, whey systems show the expected acidic coacervation window, whereas RP remains generally turbid with a neutral-pH maximum, consistent with glutelin-driven aggregation. Visual panels corroborate the numeric A600 trends (clearer samples reveal more of the black background; the most turbid vials appear brightest). Together, whey systems share an acidic turbidity window (pH 4–5), whereas RP shows a neutral-side maximum with a generally elevated baseline; this establishes a common pattern (pH-dependent scattering) but with protein-specific optima.

### 2.2. Rheological Characterization

#### 2.2.1. Oscillatory (Viscoelastic) Tests

**Figure 2:**
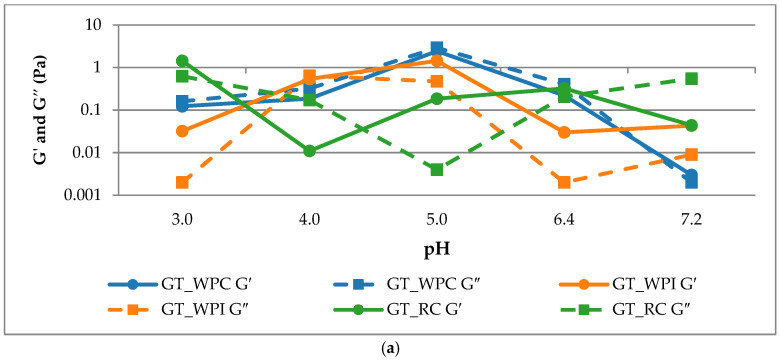

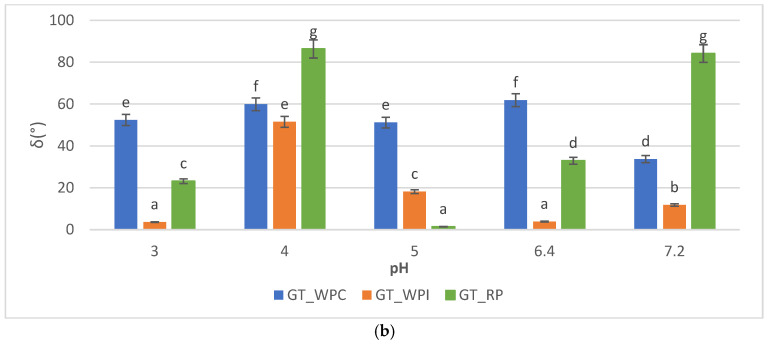
(**a**) Small-amplitude oscillatory shear (1 Hz) for GT–protein systems across pH 3.0–7.2. Combined plot of G′ (solid) and G″ (dashed) vs. pH for GT_WPC, GT_WPI and GT_RP. Measurements were performed in the linear viscoelastic regime at 1 Hz. (**b**) Phase angle (δ) of GT–protein systems (WPC80, WPI, RP) as a function of pH. Different letters above the bars indicate significant differences among pH values within each protein system.

In the present results (Table 2, Figure 2a,b) for tragacanth gum–protein systems, a clear pH dependence that accords with the mechanism of electrostatic association between proteins and an anionic polysaccharide is observed. Across proteins, a shared G′/G″ reduction away from the optimum is observed, but the optimum shifts: whey maximizes near pH 4–5 (associative window), whereas RP shows acid-side elasticity (pH ≈ 3) with a neutral-side viscosity rise, reflecting solubility-driven clustering.

Both whey systems exhibit a maximum in elasticity around pH 5, but with different characters. In WPC, the peak at pH 5 (G′ = 2.428 and G″ = 2.918 Pa) occurs with G″ > G′ (δ ≈ 52°), indicating a viscous-dominated, soft flocculated network despite the high G′. In WPI, the pH-5 maximum (G′ = 1.440 Pa and G″ = 0.472 Pa) shows that G′ > G′′ (δ ≈ 18°) indicated a clearly gel-like, solid-dominated structure. Outside this window (pH 3–4 and 6.4–7.2), both whey systems display marked decreases in G′ and G″, consistent with network loosening. At very low moduli (e.g., WPI pH 3 and 6.4), the small δ reflects a weak, easily disrupted gel. Complexes with rice protein (RP) follow a different pattern. The highest elasticity at pH 3 (G′ = 1.431 Pa; δ ≈ 23°) indicates a weak but elastic gel, whereas pH 4 and 7.2 are distinctly liquid-like (δ ≈ 86° and 84° with low G′). A secondary, modest rise appears at pH 5–6.4 (G′ ≈ 0.19–0.32 Pa; δ ≈ 33°). Throughout, we classify “solid-like” as tan δ = G′′/G′ < 1 (δ < 45°) and “liquid-like” as tan δ > 1 (δ > 45°) [21]. Superscript letters denote significant differences within each column (ANOVA/Tukey), confirming that the pH-5 condition is uniquely high for the whey systems in their respective dominant moduli, while RP shows an acid-side maximum and minimal structure at neutral pH. Consistent with the steady-shear analysis, whey systems show their strongest structure within the acidic window, whereas the rice–protein system exhibits an elastic maximum on the acid side (pH 3) but a viscosity maximum closer to neutral pH, reflecting different sensitivities of SAOS versus steady-shear to network elasticity and dissipative clustering.

#### 2.2.2. Steady-Shear Flow Behaviors

**Figure 3:**
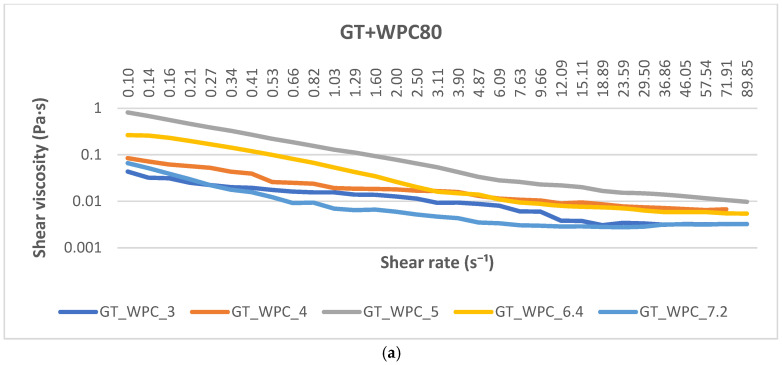

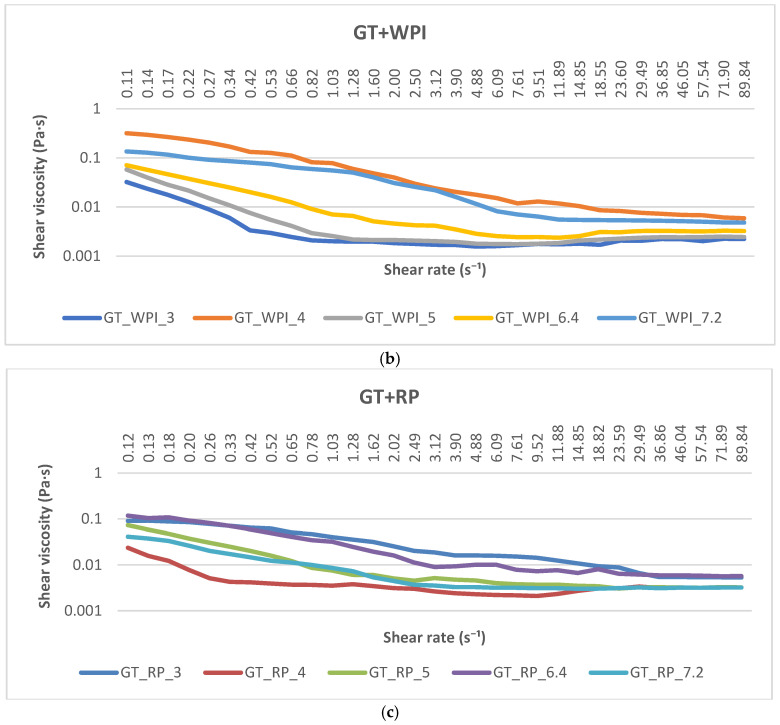
Log–log plots of apparent viscosity η versus shear rate (s^−1^) for GT + WPC80 (**a**), GT + WPI (**b**), and GT + RP (**c**) at 22 °C and pH 3.0–7.2. Colors denote pH; axis limits are identical across panels.

Across all three systems (Figure 3), the flow curves exhibit pronounced shear-thinning over ~10^−1^ to 10^2^ s^−1^ with no extended high-shear Newtonian plateau, consistent with deformable, breakable transient networks reported for protein–polysaccharide complexes [22,23]. For GT + WPC, apparent viscosity is highest at pH 5, followed by pH 6.4, and drops markedly at pH 3–4 and at pH 7.2. This ordering indicates that the most space-spanning structure forms close to the known complexation window for whey proteins with anionic polysaccharides, where attractive interactions are strongest and transient aggregates resist flow before being eroded by shear. For GT + WPI, the curves are led by pH 4 (highest), followed by pH 7.2; pH 6.4 is intermediate, and pH 5 and pH 3 are lowest. The maximum at mildly acidic pH (≈4) is consistent with associative complexation near the whey protein pI, whereas the secondary rise at neutral pH (7.2) likely reflects segregated structuring and/or aggregate growth under these conditions, which increases light scattering and low-shear viscosity even as purely electrostatic attraction weakens. In contrast, moving to pH 5 and 3 yields lower viscosity, indicating less extensive, more easily disrupted structures under steady-shear. For GT + RP, the highest viscosity is observed at pH 6.4, with pH 3 also elevated, whereas pH 4, 5 and pH 7.2 are comparatively lower. This profile is consistent with the solubility/aggregation landscape of glutelin-rich rice proteins: improved solubility and partial unfolding near neutral pH increase the hydrodynamic volume and promote weak, shear-breakable clusters with GT, while at strongly acidic pH (~3), stronger electrostatic attraction also raises viscosity but yields a more brittle network under flow. In Figure 3, this “brittle-under-flow” behavior manifests as a steeper negative slope of apparent viscosity as a function of shear rate at the pH extremes (e.g., WPC80 pH 3–4, 7.2; WPI pH 3, 6.4; RP pH 3), with an early collapse to low η by ~10–20 s^−1^, whereas the pH of maximum structure maintains higher η over the same shear-rate range. Analysis was restricted to the shear-rate range where torque and normal-force signals remained stable and the free surface was intact. No signatures of edge fracture were observed within the reported range (no spiking in torque or normal force and no visible meniscus recession). The low-shear viscosity rankings (η at ≈ 0.1–0.2 s^−1^) are broadly consistent with the SAOS trends. For GT + WPC80, the highest η at pH 5 mirrors the SAOS maximum in moduli at pH 5. For GT + WPI, η peaks at pH 4, while the largest G′ occurs at pH 5, indicating that at pH 4, dissipative contributions (G″ comparable to G′) increase low-shear resistance even though the elastic network is weaker. For GT + RP, η is the highest near pH 6.4 (with pH 3 also elevated), whereas SAOS elasticity peaks at pH 3. This divergence agrees with a picture in which increased solubility and hydrodynamic volume near neutral pH raise steady-shear viscosity, while strongly acidic conditions produce a more elastic but brittle network under flow.

### 2.3. Particle Size by Dynamic Light Scattering (DLS)

**Figure 4:**
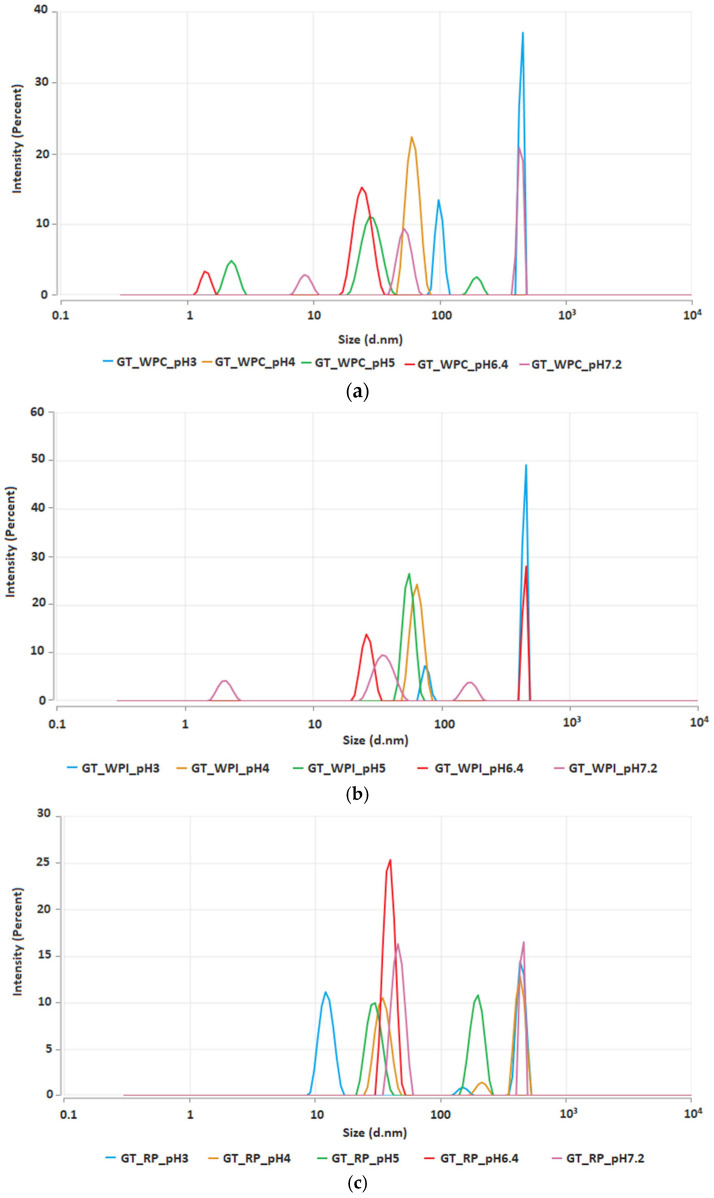
Intensity-weighted hydrodynamic size distributions measured by DLS. (**a**) GT + WPC, (**b**) GT + WPI, (**c**) GT + RP at pH 3, 4, 5, 6.4 and 7.2.

Across all three GT–protein systems, the intensity-weighted profiles are multimodal and pH-dependent. Because intensity weighting in DLS scales approximately with d^6^, even a small number of large clusters strongly biases the signal. That is why the reported modes are interpreted as relative intensity peaks rather than number fractions [24]. For the complex GT + WPC (Figure 4a) at pH 5–6.4, the main (highest) peaks lie below 100 nm (≈20–60 nm). At pH 4, the main peak is ~80–100 nm. At pH 3 and pH 7.2, the distributions shift to ~10^3^ nm, with only minor sub-100 nm contributions. For the complex GT + WPI at pH 4–5, the main peaks are sub-100 nm (≈60–90 nm). At pH 6.4, a narrow peak appears at ~30–50 nm. At pH 3, a pronounced ~10^3^ nm peak dominates. At pH 7.2, the main peak lies in 10–100 nm (≈60–90 nm), accompanied by a weak shoulder > 100 nm and a minor 1–10 nm mode. No strong micron-scale peak is observed. In GT + RP complex, at pH 6.4, the main peak is ~80–100 nm. At pH 5, the distribution is bimodal (~50–70 nm plus ~150–200 nm). At pH 3, a ~20–30 nm peak is present. At pH 4, there are two clear peaks: ~60–80 nm and a strong ~10^3^ nm peak. At pH 7.2, the main peak is again ~60–80 nm, with a weaker sub-micron/≈10^3^ nm shoulder. The DLS trends qualitatively align with the rheology measured at the same pH. All systems exhibit multimodal, intensity-weighted distributions, yet the dominant modes differ: sub-100 nm nanocomplex prevalence coincides with the whey acidic window, while larger clusters contribute to near-neutral pH for RP; thus, DLS confirms shared pH sensitivity with protein-specific dispersion states. For GT + WPC80, broader/larger apparent sizes coincide with the viscosity maximum at pH 5 and elevated viscoelastic response. For GT + WPI, increased sizes around pH ≈ 4 match the higher low-shear viscosity; and for GT + RP the largest sizes near pH 6.4 agree with the steady-shear viscosity maximum. Cross-plots of η(0.1 s^−1^) and G′(1 Hz) versus DLS Z-average illustrate a positive association for the whey systems and a partial divergence for rice protein, consistent with the stronger role of network connectivity under flow compared with isolated-cluster size captured by DLS.

### 2.4. FTIR Analysis

All spectra are shown for 1800–900 cm^−1^ and were baseline-corrected and normalized to the amide-I maximum to allow comparison across pH. Spectrally, all proteins share stable amide I/II positions with modest shifts, while GT-related bands (COO^−^ ~1446–1400 cm^−1^; 1175–1000 cm^−1^) strengthen or sharpen most clearly within the whey acidic window and shift in relative prominence near neutral pH for RP, supporting the two-regime interaction pattern inferred from turbidity, rheology, and DLS. A prominent amide-I band is visible in every trace at ~1622–1633 cm^−1^ and an amide-II band between ~1516 and 1539 cm^−1^ in aqueous ATR these lie close to the H-O-H bending region (water, ~1640–1650 cm^−1^), so differences are reported as relative positions and intensities. In the polysaccharide fingerprint region (~1175–1000 cm^−1^), clear bands appear near 1446–1400 cm^−1^ (COO^−^/CH_2_) and across 1259–1000 cm^−1^, with resolved features around ~1170, ~1077, ~1039–1018 and, in several cases, ~1006 cm^−1^. It was also noticed that the feature at ~1259 cm^−1^ belongs to amide III rather than the GT fingerprint.

**Figure 5:**
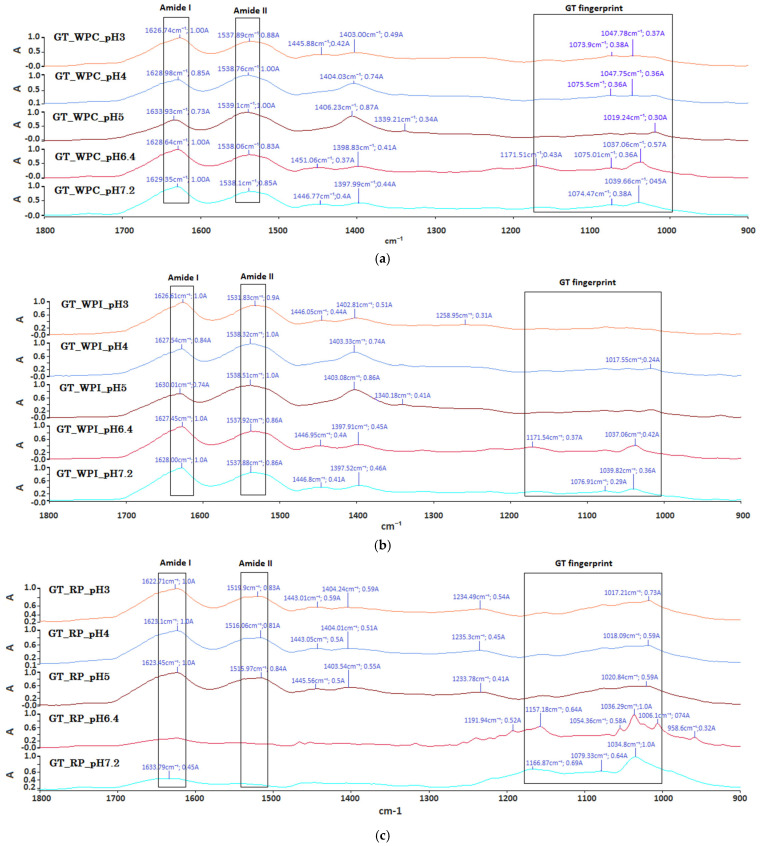
FTIR spectra of tragacanth gum (GT)–protein mixtures across pH 3–7.2: (**a**) GT + WPC80, (**b**) GT + WPI, (**c**) GT + RP (1800–900 cm^−1^; traces vertically offset).

For GT + WPC (Figure 5a), the amide-I maximum appears at 1626.7 cm^−1^ (pH 3), 1629.0 cm^−1^ (pH 4), 1633.9 cm^−1^ (pH 5), 1628.3 cm^−1^ (pH 6.4) and 1629.4 cm^−1^ (pH 7.2), while amide-II is found at 1537.9, 1538.8, 1539.1, 1537.9 and 1538.1 cm^−1^, respectively. A band near 1446–1451 cm^−1^ is clearly resolved at pH 3 (1445.9 cm^−1^), pH 6.4 (1451.1 cm^−1^) and pH 7.2 (1446.8 cm^−1^), whereas at pH 4 and pH 5, it is not resolved as a distinct peak. The ~1400 cm^−1^ region is present at all pH (1403.0–1406.2 cm^−1^ for pH 3–5; 1398.8–1398.0 cm^−1^ for pH 6.4–7.2). In the fingerprint window, features near 1073–1075 cm^−1^ appear at pH 3–4 and again at pH 6.4–7.2, peaks at ~1047–1048 cm^−1^ are evident at pH 3–4, peaks around 1037–1040 cm^−1^ occur at pH 6.4–7.2, and a band at 1019.2 cm^−1^ is observed only at pH 5. For GT + WPI (Figure 5b), the amide-I maximum lies at ~1626–1630 cm^−1^ for all pH with variations ≤ 4 cm^−1^, and amide-II appears between ~1532 and 1538 cm^−1^. A band near ~1446 cm^−1^ is clearly resolved at pH 3, 6.4 and 7.2, whereas at pH 4 and 5, it is not resolved as a distinct peak. In the fingerprint region, a band near ~1259 cm^−1^ (amide III) is visible at pH 3. A distinct feature appears close to ~1018 cm^−1^ at pH 4; and for pH 6.4–7.2, the bands around ~1172, ~1077 and ~1038 cm^−1^ are present with lower relative intensity. For GT + RP (Figure 5c), the amide-I maximum remains near ~162–1624 cm^−1^ from pH 3 to 6.4 and shifts up to ~1633–1634 cm^−1^ at pH 7.2, while amide-II occurs at the lower end of the observed range (~1516–1520 cm^−1^). Within 1446–1400 cm^−1^, the ~1446 and ~1403–1404 cm^−1^ bands are present at all pH with modest intensity changes, and the 1175–1000 cm^−1^ window shows marked pH-dependence: the ~1036 cm^−1^ band is most intense at pH 6.4, whereas at pH 7.2, the ~1167 and ~1079 cm^−1^ bands are relatively stronger and the ~958–1006 cm^−1^ region is weaker. Because spectra were normalized to the amide I maximum, the polysaccharide fingerprint (1175–1000 cm^−1^) appears relatively flat in the WPI series: protein absorption dominates and GT bands are inherently broad/overlapping. This does not indicate the absence of GT signals, rather, it reflects lower relative amplitude and less ordering outside the acidic window. Sharpening of the ~1017–1020 cm^−1^ feature at pH 4–5 remains consistent with enhanced electrostatic pairing.

### 2.5. Molecular Docking with AutoDock Vina

Local binding between tragacanth segments and protein surfaces (WPC80, WPI, RP) was probed using AutoDock Vina 1.1.2 via the CB-Dock2 web server (https://cadd.labshare.cn/cb-dock2/index.php; accessed 15 September 2025), which searches ligand poses and ranks them by an empirical estimate of binding free energy (ΔG, kcal·mol^−1^); more negative scores indicate more favorable local interactions, although absolute energies are approximate and are best used to assess within-protein trends (differences < ~1 kcal·mol^−1^ are not considered significant). Visualization as surface hydropathy maps highlights cationic patches (Lys/Arg) and polar H-bonding neighborhoods that promote pairing with GT carboxylates, whereas hydrophobic/aromatic grooves (Phe/Tyr/Trp) can provide additional anchoring via CH-π/hydrophobic contacts [25].

**Figure 6:**
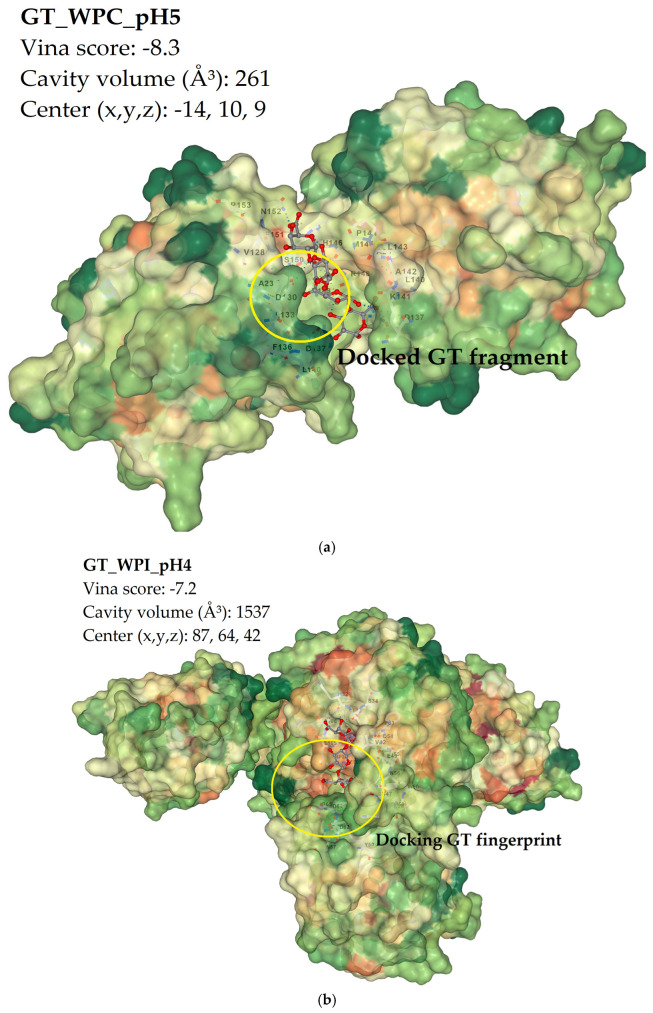

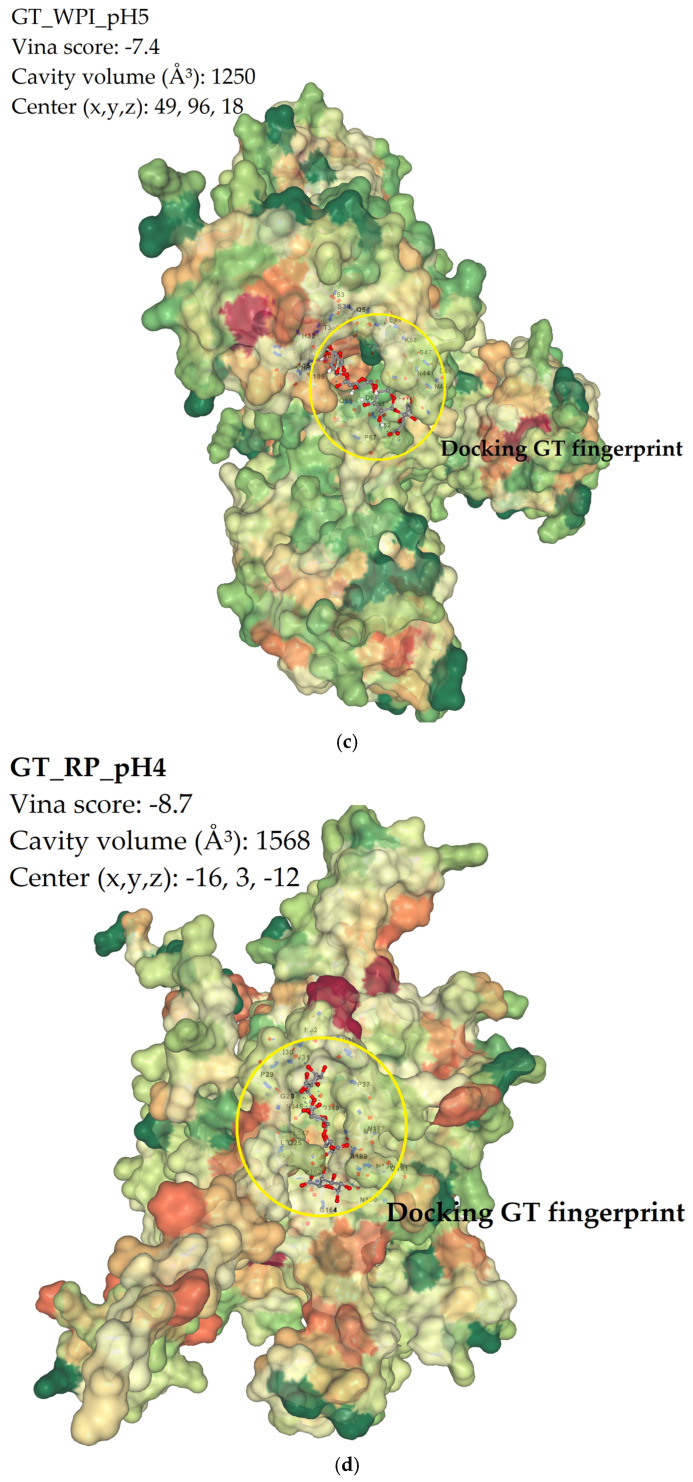

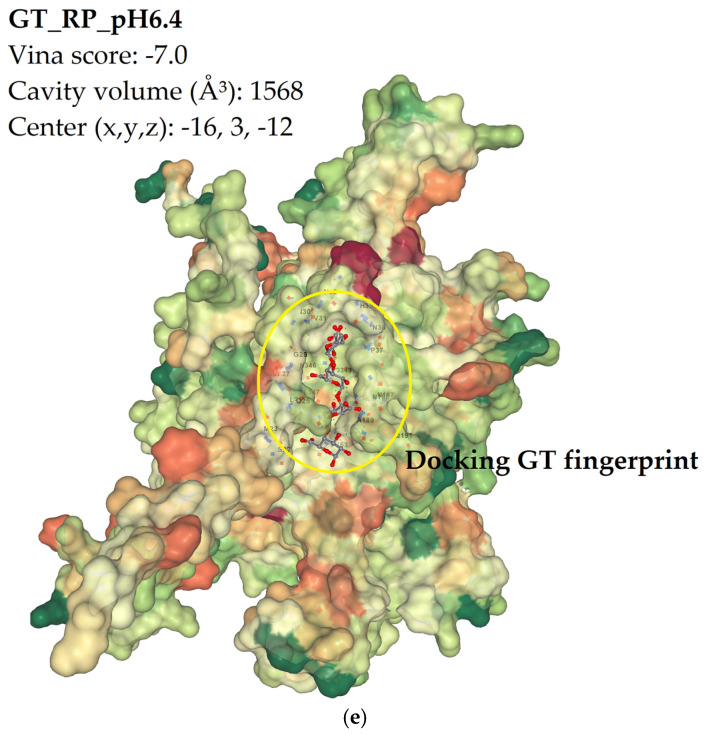
Representative AutoDock Vina poses of tragacanth gum (GT) on protein surfaces, shown as hydropathy-colored molecular surfaces: (**a**) GT-WPC80, pH 5 (best Vina score within WPC80), (**b**) GT-WPI, pH 4 (acidic coacervation window), (**c**) GT-WPI, pH 5 (acidic window), (**d**) GT-RP, pH 4 (most favorable Vina score for RP), and (**e**) GT-RP, pH 6.4 (condition of viscosity/DLS maximum). Surface colors denote hydropathy: dark green = more hydrophobic, white/cream = intermediate, light orange = more polar; GT is shown as sticks colored by element, and the yellow ring marks the detected pocket. The overlaid “Docking GT fingerprint” box marks the local GT–protein contact zone. Surfaces highlight hydrophobic/hydrophilic patches, and the GT fragment is shown as sticks. Dashed lines indicate example H-bonds, and nearby Lys/Arg and Asp/Glu labels mark potential salt-bridge sites with GT–COO^−^. All panels use a fixed viewing angle and scale for comparability.

The docking summary (Table 3) reports Vina scores, cavity volumes, and grid centers for each GT–protein-pH condition. For GT + WPC, the scores span −7.4 to −8.3 kcal·mol^−1^ with the most favorable value at pH 5 (−8.3); the sampled cavities for WPC are comparatively small (mostly ~261 Å^3^, with a single larger site of ~400 Å^3^ at pH 4). For GT + WPI, the scores lie between −7.2 and −8.0 kcal·mol^−1^ across pH, while the detected cavities are consistently large (≈1250–1537 Å^3^). For GT + RP, the scores range from −7.0 to −8.7 kcal·mol^−1^, the most favorable appearing at pH 4 (−8.7), with cavity volumes ~1050–1568 Å^3^. Figure 6a shows the representative pose for GT + WPC at pH 5, corresponding to the best WPC score in the table. The fragment is docked in a shallow surface groove bordered by mixed hydrophilic and hydrophobic patches; the pose sits within the smaller-volume pocket typical of WPC in Table 3. Figure 6b displays GT + WPI at pH 4 and Figure 6c GT + WPI at pH 5; in both panels, the ligand occupies a broader cleft on the protein surface, consistent with the larger cavity volumes reported for WPI. The mapped surfaces around the pose contain contiguous polar regions with nearby basic and acidic side-chain neighborhoods as well as hydrophobic patches visible along the groove. Figure 6d presents GT + RP at pH 4, which corresponds to the lowest (most favorable) GT + RP score in Table 3; the ligand is positioned inside a deep pocket on the RP surface with an extended contact footprint. Figure 6e shows GT + RP at pH 6.4; the pose is located in a similarly sized pocket to that reported in the table for this condition, with surface coloring indicating adjacent polar and hydrophobic areas.

## 3. Discussion

Across the three GT–protein systems, a coherent, pH-selective picture emerges from all techniques. Across all readouts, a common, pH-dependent response is observed (changes in turbidity, viscoelasticity, and dispersion), but the location and character of the optimum are protein-specific: whey aligns with an associative acidic window (pH ~4–5), whereas RP exhibits solubility/aggregation-controlled behavior centered closer to neutrality, with an acid-side elastic peak and a neutral-side viscosity maximum. In the whey systems, an acidic window is consistently observed: turbidity rises, the storage modulus reaches a maximum, and DLS is dominated by sub-100 nm modes in the same pH range. This pattern matches the established view that electrostatic complexation and, where dense enough, complex coacervation between globular whey proteins and anionic polysaccharides are strongest near the protein isoelectric region (≈4.5–5.5) [4,18,26,27,28,29]. In this regime, positively charged patches on the protein surface pair with GT carboxylates, while multiple hydrogen bonds and local hydrophobic contacts stabilize many small associative complexes that percolate into a weak gel network. FTIR measurements support this picture. Within the acidic window, relative strengthening is observed in the COO^−^ and carbohydrate bands (≈1446–1400 and 1175–1000 cm^−1^), consistent with increased polysaccharide–protein contacts, whereas changes in Amide I/II remain modest, as expected for measurements where Amide I overlaps the H–O–H bending band.

The non-coincidence between the maxima of G′ and apparent viscosity is informative rather than contradictory. Small-amplitude oscillation probes the strength of the undisturbed network, which is tuned by electrostatic complexation. Steady-shear emphasizes how readily those complexes break and align. Consequently, the pH of maximum viscosity can be shifted relative to the pH of maximum G′ by a fraction of a pH unit, as we observe in presented research. WPC80 shows its largest viscosities around pH 5–6.4, whereas in WPI, the viscosity peaks at pH 4 and displays a secondary rise at pH 7.2. The neutral-pH shoulder in WPI, seen in turbidity and flow, but not accompanied by a strong elastic response, is compatible with segregated structuring and/or growth of mid-size aggregates under the chosen ionic strength, which intensify low-shear viscosity and light scattering without the tighter associative binding evident in the acidic window [19,20,26,28]. Whey + GT networks are most cohesive near pI, while near neutral pH, the system behaves more as a viscous, shear-breakable dispersion.

Rice protein behaves differently, in line with its composition and solubility landscape. Reviews emphasize that native RP, dominated by glutelin, has low aqueous solubility and a high aggregation propensity throughout pH 4–7.2, improving mainly under extreme pH or after modification [30,31,32,33]. This accounts for the generally high turbidity which was observed and for the neutral-side viscosity maximum at pH 6.4, despite a strong oscillatory signal already at pH≈3. Our DLS shows that near pH 6.4, the distributions are enriched in ~80–100 nm modes (with components up to ~150–300 nm), indicative of numerous mid-size clusters that are readily broken by shear, yet sufficient to elevate apparent viscosity. At very acidic pH, the positive charge on RP increases and GT carries higher charge density, which favors cleaner electrostatic complexes and a higher G′. However, such networks are brittle and shear-thin rapidly, so the viscosity does not necessarily reach the neutral-pH level. The resulting split between the maxima of G′ and η is, therefore, mechanistically consistent with association-dominated elasticity versus shear-dominated flow in mixed biopolymer systems and emulsion-filled or associated gels [34,35].

Interpreting the DLS correctly is crucial. Intensity-weighted size distributions over-represent rare, large particles because Rayleigh scattering scales approximately with the sixth power of diameter. It is, therefore, expected that a small fraction of very large clusters at extreme pH can dominate A600 while the population remains governed by nanocomplexes. This is exactly what we see: in whey systems, the acidic window exhibits abundant sub-100 nm modes alongside elevated G′, whereas at extreme pH (e.g., WPI at pH 3), the appearance of ~10^3^ nm modes rationalizes strong light scattering and easy yielding under shear [24,36,37]. When combined with FTIR, these distributions reinforce a simple split: tightened carbohydrate/COO^−^ bands and nanocomplex prevalence in the mildly acidic window versus broadened/split carbohydrate features and larger-cluster modes away from it [26,27].

Molecular docking plays a complementary, local role. Vina scores for WPC cluster around −7.4 to −8.3 kcal·mol^−1^, with the most favorable value at pH 5. WPI shows comparable scores across pH (−7.2 to −8.0) but in larger surface cavities. RP presents its most favorable score at pH 4, while other pH values lie between −7.0 and −7.6. Given the qualitative nature of docking and the limited discriminating power of score differences below ~1 kcal·mol^−1^, these results are best read as maps of likely multivalent “hot-spots”, rather than thermodynamic rankings. The representative poses reveal neighborhoods rich in Lys/Arg (potential salt bridges with GT-COO^−^), donors/acceptors for hydrogen bonding, and adjacent hydrophobic or aromatic grooves that can contribute additional anchoring [25,38,39,40,41]. This helps explain why several pH points display similar local affinities while their bulk responses diverge. DLS, turbidity and rheology depend not only on local contacts but also on solubility, dispersion state and aggregation kinetics at nano- to micrometer scales.

Viewed together, the data offer a practical handle for formulation. In whey + GT systems, targeting pH ≈ 4–5 yields the most cohesive, gel-like behavior and elevated turbidity, as is widely reported for whey–anionic-polysaccharide complexes [10,11,12,13,26,27,28,29]. Moving toward neutrality reduces cohesion and increases viscous, shear-breakable character, which can be useful when high viscosity with limited “gel strength” is desired. In RP + GT, the broad turbidity baseline and the maximum viscosity at pH ~6.4 with relatively modest elasticity suggest applications that seek thickening without strong gelation. Literature indicates that pH shift or processing can further improve RP solubility and expand this neutral-side window [30,31,32].

Compared with prior protein–gum reports that focused largely on single proteins and bulk rheology, the present work maps protein-dependent, pH-selective windows across three protein classes (WPC80, WPI or rice protein) and links molecular signatures to dispersion state and mechanics [27,28]. The acidic window in whey is established not only by viscosity or G′ but also by convergent FTIR markers (COO^−^ and 1015–1040 cm^−1^ tightening) and DLS evidence for sub-100 nm nanocomplexes, providing a molecular rationale for why elasticity peaks near the isoelectric region [4,27,28,42,43]. Conversely, the neutral-side shoulder in WPI and the pH 6.4 maximum in rice protein reveal segregative/solubility-controlled aggregation outside the associative window-behavior that bulk rheology alone cannot unambiguously distinguish [4,20,35,44]. Finally, docking acts as a qualitative map of multivalent hot-spots (Lys/Arg patches, H-bonding neighborhoods, adjacent hydrophobic grooves), explaining how similar local affinities can yield different bulk outcomes depending on solubility and cluster growth [25,38,39,44]. Together, these results deliver actionable formulation ranges (≈ pH 4–5 for cohesive GT–whey networks, a neutral-side maximum centered near pH 6.4 for high-viscosity GT–RP systems) and a molecularly anchored framework that advances beyond descriptive rheology [20,27,28,29,35,42,43,45,46].

## 4. Materials and Methods

### 4.1. Materials

Whey protein concentrate (WPC80) was obtained from Mlekovita (Wysokie Mazowieckie, Poland), whey protein isolate (WPI) from Carbery (Ballineen, Republic of Ireland), and rice protein (RP) from Beneo (Mannheim, Belgium). Tragacanth gum (GT) was purchased from Food Colours (Piotrków Trybunalski, Poland). The buffering agent PIPES and HEPES was acquired from Pol-Aura (Olsztyn, Poland), and glacial acetic acid (≥99.5%) from POCH S.A. (Gliwice, Poland). All other chemicals and reagents used in the study were of analytical grade. Deionized water was used throughout all experiments.

### 4.2. Preparation of Stock Solutions

Tragacanth gum (TG) and proteins (WPC80, WPI, or RP) were separately dissolved in selected buffer systems (pH 3.0, 4.0., 5.0, 6.4 or 7.2) at final concentrations of 0.2% (*w*/*v*) for TG and 2.0% (*w*/*v*) for proteins. Both TG and protein powders were dispersed at room temperature (22 °C) under continuous magnetic stirring (300 rpm) for 2 h and then stored at 4 °C overnight to ensure complete hydration. After hydration, TG and protein stock solutions were mixed in a 1:1 (*v*/*v*) ratio to obtain final concentrations of 0.2% (*w*/*v*) GG and 2.0% (*w*/*v*) protein. The mixtures were gently stirred at room temperature for 10 min and then stored at 4 °C overnight to allow complex formation under cold-set conditions (Table 4).

### 4.3. Turbidity Measurements

The optical density (OD) was measured at 600 nm using a UV–Vis spectrometer (Evolution 201 UV-Visible, Thermo Fisher Scientific, Waltham, MA, USA) at 22 °C. Quartz cuvettes with optical path length L = 1 cm (working volume 4.0 mL) were used. Readings were taken against buffer blanks matched to each sample’s pH and ionic strength. For each sample, n = 3 technical replicates were recorded and reported as mean ± SD. Values ≥ A600 1.5 were noted as outside the linear range and interpreted semi-quantitatively for within-series comparisons.

### 4.4. Visual Appearance

Sample appearance was documented to support turbidity trends. For each protein–tragacanth mixture, the pH series (3, 4, 5, 6.4, 7.2) was arranged left-to-right from lower-to-higher pH and photographed at 22 °C against a matte black background under uniform diffuse illumination. System identity (WPC, WPI, RP) and pH order are indicated in the figure. The assembled panel is provided as Figure 1.

### 4.5. Rheological Characterization

Rheological tests were carried out on a rotational rheometer Kinexus lab+ (Malvern Panalytical, Cambridge, UK) fitted with serrated plate–plate geometries (upper PU40X SW1382 SS; lower PLS40X S2222 SS) to suppress wall slip. Data were recorded with rSpace. Samples were tested in triplicate at 22 °C; the gap was 1 mm and plates were cleaned and re-zeroed between runs.

#### 4.5.1. Oscillatory (Viscoelastic) Tests

Oscillatory measurements were conducted in small-amplitude oscillatory shear at 1 Hz, using a low strain chosen to avoid non-linear effects, at 22 °C. Storage (G′), loss (G″) moduli and tan δ were recorded. Oscillatory tests were performed at 1 Hz within the linear viscoelastic regime. No data above the instrument’s reliable frequency range (e.g., ~tens of Hz) were analyzed to avoid inertial/compliance artifacts in G′ and G″. Steady-shear viscosity was measured as a function of shear rate (0.1–100 s^−1^) at 22 °C. All tests were performed in triplicate.

#### 4.5.2. Steady-Shear Flow Behavior

Shear-stress (τ) data were exported, and apparent viscosity was calculated as as the ratio of shear stress to shear rate; points were log-spaced from 0.12 to 90 s^−1^, and for each point, the last 80% of the time window was averaged. Three runs were independently conducted for every formulation; at every shear-rate, we calculated mean ± SD and excluded values below the torque resolution of the instrument or showing transient artifacts. Curves were graphed as η (Pa·s) vs. shear-rate (s^−1^), with the same color/label for the same sample between samples; individual replicates were graphed as thin lines and the mean as a thicker overlay. To reduce artifacts at high shear rates in plate–plate geometry, samples were trimmed flush with the plate edge, equilibrated at the target gap prior to each sweep, and the free surface was visually inspected throughout the measurements. Torque and normal force were continuously monitored. Data showing spikes/oscillations or any visible meniscus recession were excluded. Accordingly, interpretation is restricted to the shear-rate range where signals remained stable, and the free surface remained intact.

### 4.6. Particle Size by Dynamic Light Scattering (DLS)

Hydrodynamic size was measured on a Zetasizer Ultra (Malvern Panalytical) using non-invasive backscatter detection (173°). Samples were run in disposable sizing cuvettes (DTS0012) at 22 °C with 120 s equilibration; data were analyzed with the instrument’s General Purpose model. Dispersant properties were set to the measurement medium: water (RI = 1.333; η ≈ 0.955 mPa·s at 22 °C) or the matching buffer at the sample pH. Intensity correlation functions were saved for quality control. Each condition was prepared independently and measured in triplicate. Results are reported as intensity-weighted size distributions from single-angle DLS.

### 4.7. FTIR Analysis

Spectra were collected on a PerkinElmer FT-IR/NIR Spectrum 3 in transmission mode using IR-transparent liquid cells (CaF_2_). Aqueous GT–protein samples at pH 3.0, 4.0, 5.0, 6.4, or 7.2 were measured at 22 ± 1 °C over 4000–600 cm^−1^ with 4 cm^−1^ resolution and 16–32 co-added scans. For each pH, a matching background spectrum (buffer at the same pH) was recorded immediately before the sample. Spectra were baseline-corrected and, where indicated, normalized to Amide I.

### 4.8. Molecular Docking

Molecular docking was conducted to examine interactions between tragacanth-derived carbohydrate oligomers and three protein systems representative of food matrices. As receptors, whey β-globular protein was used to represent WPC-type material, a whey α-lactalbumin protein to represent WPI-type material (retaining the native divalent cation), and a rice 11S-type storage globulin (glutelin) obtained as a computed 3D model from a public structure repository. Experimental pH effects were modeled by preparing five protonation states (pH 3.0, 4.0, 5.0, 6.4 or 7.2) for each receptor and for the ligand.

Protein protonation states were assigned with a standard, structure-based empirical pKa workflow (PROPKA-class approach) using an AMBER-family force field and hydrogen-bond network optimization; crystallographic waters were removed prior to docking. In parallel, the carbohydrate ligand (galacturonic acid–rich oligomer) was generated in matching pH-specific ionization states using an open-source cheminformatics toolkit that applies pH-dependent protonation rules for weak acids; output was saved in a format preserving atom types and partial charges. For every condition, receptor and ligand with the same pH were paired (pH-matched docking). Docking employed an automated cavity-guided platform that detects plausible binding pockets, constructs local search boxes, and evaluates poses with a Vina-type scoring function under default settings. For each run, the top-ranked pose (most negative score) was retained. From the pocket report we recorded the docking score (kcal mol^−1^), cavity volume (Å^3^), and pocket center coordinates (x, y, z). It extracted contact residues to compare qualitative interaction patterns across pH. Figures were prepared directly from the browser-based 3D viewer associated with the docking platform by rendering receptors as molecular surfaces colored by hydrophobicity and ligands in ball-and-stick style; identical viewing parameters were applied across panels to enable direct visual comparison.

### 4.9. Statistic

Data were analyzed using STATISTICA 12.0 PL (StatSoft Polska Sp. z o.o., Kraków, Poland). A three-way ANOVA evaluated main and interaction effects of protein type (WPC, WPI or RP), pH (3, 4, 5, 6.4, 7.2), and tragacanth gum concentration on each measured variable. Multiple comparisons were conducted with Tukey’s post hoc test at *p* < 0.05.

## 5. Conclusions

Protein type and pH jointly set structure–function windows in tragacanth–protein systems. In whey, a mildly acidic window (~pH 4–5) characterizes an associative regime near the isoelectric region. Within this window, higher turbidity, elevated storage modulus (G′), and sub-100 nm DLS modes co-occur, consistent with electrostatically driven association. WPI additionally shows a neutral-pH shoulder (pH 7.2) in turbidity/viscosity without a matching rise in G′, which is consistent with segregated, solubility-led aggregate growth rather than network strengthening. Rice protein differs: a solubility/aggregation-controlled neutral-side regime (broadly elevated turbidity and a viscosity/DLS maximum at ~pH 6.4) contrasts with a modest acid-side increase in G′ (~pH 3) that reflects local contacts that shear-thin readily, explaining the non-coincidence of G′ and η peaks. Method readouts are complementary: small-amplitude oscillatory tests capture undisturbed network strength, steady-shear emphasizes breakup/alignment, and DLS, while intensity-weighted, clarifies dispersion state. FTIR highlights the informative 1700–900 cm^−1^ window and corroborates stronger coupling at pH 3–5, and docking maps multivalent hot-spots that rationalize how similar local affinities can yield different bulk responses. Together, these findings define actionable formulation ranges—~pH 4–5 for cohesive whey + GT networks and ~pH 6–7 for high-viscosity RP + GT systems—and provide a concise molecular-to-bulk rationale for tuning texture with tragacanth. Scope and limitations: conclusions apply to the tested compositions, ionic strength, and temperature; generalization beyond these conditions should be made with caution. Outlook: future work should map protein level and salt effects, processing history, and protein modifications (especially for rice protein) to broaden neutral-pH functionality and validate the robustness of the identified windows.

## Figures and Tables

**Table 1:** Turbidity (A600) of GT–protein mixtures at different pH values (3.0–7.2).

Sample ID	Turbidity ± SD
GT_WPC_pH3	0.835 ^a^ ± 0.008
GT_WPC_pH4 *	2.404 ^f^ ± 0.002
GT_WPC_pH5 *	1.956 ^e^ ± 0.004
GT_WPC_pH6.4 *	1.734 ^d^ ± 0.018
GT_WPC_pH7.2	1.492 ^c^ ± 0.003
GT_WPI_pH3	0.728 ^a^ ± 0.006
GT_WPI_pH4 *	1.998 ^e^ ± 0.003
GT_WPI_pH5	1.493 ^c^ ± 0.015
GT_WPI_pH6.4	0.938 ^a^ ± 0.008
GT_WPI_pH7.2 *	2.121 ^e^ ± 0.004
GT_RP_pH3	0.891 ^a^ ± 0.036
GT_RP_pH4 *	1.529 ^c^ ± 0.047
GT_RP_pH5	1.392 ^b^ ± 0.005
GT_RP_pH6.4	1.270 ^b^ ± 0.089
GT_RP_pH7.2 *	2.368 ^f^ ± 0.041

* Indicates A600 ≥ 1.5; such values were interpreted as semi-quantitative due to potential multiple scattering; ^a–f^ different superscripts within a column indicate significant differences among means (*p* < 0.05; Tukey’s HSD); SD—standard deviation; for whey proteins, the acidic window occurs at pH 4–5 (mark in the table).

**Table 2:** Small-amplitude oscillatory shear parameters (G′, G″, δ) of tragacanth gum (GT)–protein systems (WPC80, WPI or rice protein) as a function of pH (3–7.2).

Sample ID	G′ (Pa) ± SD	G″ (Pa) ± SD	δ(°) ± SD
GT_WPC_3	0.123 ^ab^ ± 0.021	0.161 ^a–c^ ± 0.033	52.436 ^e^ ± 3.180
GT_WPC_4	0.185 ^ab^ ± 0.072	0.321 ^b–e^ ± 0.125	59.914 ^f^ ± 2.605
GT_WPC_5	2.428 ^d^ ± 0.986	2.918 ^g^ ± 0.554	51.181 ^e^ ± 7.824
GT_WPC_6.4	0.220 ^ab^ ± 0.039	0.407 ^c–f^ ± 0.002	61.869 ^f^ ± 2.345
GT_WPC_7.2	0.003 ^a^ ± 0.002	0.002 ^a^ ± 0.002	33.691 ^d^ ± 3.958
GT_WPI_3	0.032 ^a^ ± 0.003	0.002 ^a^ ± 0.0001	3.582 ^a^ ± 0.501
GT_WPI_4	0.537 ^b^ ± 0.205	0.640 ^f^ ± 0.130	51.502 ^e^ ± 5.861
GT_WPI_5	1.440 ^c^ ± 0.123	0.472 ^d–f^ ± 0.142	18.151 ^c^ ± 6.991
GT_WPI_6.4	0.030 ^a^ ± 0.003	0.002 ^a^ ± 0.0002	3.811 ^a^ ± 0.457
GT_WPI_7.2	0.043 ^a^ ± 0.005	0.009 ^a^ ± 0.004	11.803 ^b^ ± 3.828
GT_RP_3	1.431 ^c^ ± 0.194	0.623 ^f^ ± 0.183	23.125 ^c^ ± 3.852
GT_RP_4	0.011 ^a^ ± 0.007	0.172 ^a–c^ ± 0.029	86.342 ^g^ ± 4.404
GT_RP_5	0.185 ^ab^ ± 0.173	0.004 ^a^ ± 0.003	1.400 ^a^ ± 0.894
GT_RP_6.4	0.322 ^ab^ ± 0.013	0.208 ^a–d^ ± 0.009	32.949 ^d^ ± 0.887
GT_RP_7.2	0.044 ^a^ ± 0.022	0.548 ^e–f^ ± 0.307	84.149 ^g^ ± 7.814

^a–g^ The means in the same column with different superscripts are different significantly (*p* < 0.05, Tukey’s HSD test). Within each column, different superscript letters denote significant differences (*p* < 0.05, Tukey’s HSD). Statistical testing was conducted separately for each variable (G′, G″, δ); SD—standard deviation.

**Table 3:** AutoDock Vina docking results for tragacanth (GT) with whey protein concentrate (WPC), whey protein isolate (WPI) or rice protein (RP) across pH 3–7.2. Reported here are the top-ranked pose per condition: predicted binding free energy ΔG (kcal·mol^−1^), cavity volume (Å^3^) and grid center.

Sample ID	Vina Score *	Cavity Volume (Å^3^)	Center (x,y,z)
GT_WPC_pH3	−8.2	261	−14, 10, 9
GT_WPC_pH4	−8.0	400	−7, 13, 20
GT_WPC_pH5	−8.3	261	−14, 10, 9
GT_WPC_pH6.4	−7.4	261	−14, 10, 9
GT_WPC_pH7.2	−8.0	261	−14, 10, 9
GT_WPI_pH3	−7.7	1537	87, 64, 42
GT_WPI_pH4	−7.2	1537	87, 64, 42
GT_WPI_pH5	−7.4	1250	49, 96, 18
GT_WPI_pH6.4	−7.3	1278	49, 96, 18
GT_WPI_pH7.2	−8.0	1537	87, 64, 42
GT_RP_pH3	−7.6	1050	−1, 16, −1
GT_RP_pH4	−8.7	1568	−16, 3, −12
GT_RP_pH5	−7.1	1568	−16, 3, −12
GT_RP_pH6.4	−7.0	1568	−16, 3, −12
GT_RP_pH7.2	−7.3	1512	−16, 3, −11

* Vina scores are qualitative; differences < ~1.0 kcal·mol^−1^ are not considered significant. Single-structure docking does not capture phase behavior; results should be interpreted together with FTIR/DLS/rheology. GT, tragacanth gum; WPC80, whey protein concentrate (80%); WPI, whey protein isolate; RP, rice protein; ΔG, Vina binding energy.

**Table 4:** Sample codes and compositions of tragacanth–protein formulations prepared at different pH conditions.

Sample ID	Protein	pH
GT_WPC_pH3	Whey protein concentrate 80	3.0
GT_WPC_pH4		4.0
GT_WPC_pH5		5.0
GT_WPC_pH6.4		6.4
GT_WPC_pH7.2		7.2
GT_WPI_pH3	Whey protein isolate	3
GT_WPI_pH4		4
GT_WPI_pH5		5
GT_WPI_pH6.4		6.4
GT_WPI_pH7.2		7.2
GT_RP_pH3	Rice protein	3
GT_RP_pH4		4
GT_RP_pH5		5
GT_RP_pH6.4		6.4
GT_RP_pH7.2		7.2

GT—tragacanth gum; WPI—whey protein isolate; WPC80—whey protein concentrate; RP—rice protein.

## Data Availability

The original contributions presented in this study are included in the article. Further inquiries can be directed to the corresponding author.
